# The Dengue virus protease NS2B3 cleaves cyclic GMP-AMP synthase to suppress cGAS activation

**DOI:** 10.1016/j.jbc.2023.102986

**Published:** 2023-02-07

**Authors:** Madhurima Bhattacharya, Debipreeta Bhowmik, Yuan Tian, Huan He, Fanxiu Zhu, Qian Yin

**Affiliations:** 1 Institute of Molecular Biophysics, Florida State University, Tallahassee, Florida, USA; 2 Department of Biological Science, Florida State University, Tallahassee, Florida, USA

**Keywords:** cGAS, Dengue virus, NS3 protease, proteolytic cleavage, protein-protein interaction, cGAMP, cyclic GMP-AMP, cGAS, cyclic GMP-AMP synthase, CP-C, cleavage product C-terminal, CP-N, cleavage product N-terminal, DENV, Dengue virus, FL, full length, FP, fluorescence polarization, NS, nonstructural, SEC, size-exclusion chromatography

## Abstract

Dengue virus (DENV) is one of the most prevalent mosquito-transmitted human viruses that causes significant morbidity and mortality worldwide. To persist in the cell and consequently cause disease, DENV is evolved with mechanisms to suppress the induction of type I interferons by antagonizing cGAS-STING signaling. Using recombinant proteins and *in vitro* cleavage assays, we have shown that the DENV protease NS2B3 is capable of cleaving cGAS in the N-terminal region without disrupting the C-terminal catalytic center. This generates two major cleavage products: cleavage product N-terminal (CP-N) and cleavage product C-terminal (CP-C). We observed reduction in DNA-binding affinity of CP-C as compared to full-length cGAS. Reduction in DNA-binding affinity is also correlated with the decrease in enzymatic activity of CP-C. CP-N, on the other hand, has almost comparable DNA-binding ability as that of the full-length cGAS. In fact, CP-N competitively inhibits cyclic GMP-AMP production by both full-length cGAS and CP-C. We hypothesize that high DNA-binding affinity of CP-N enables it to sequester the DNA from CP-C and noncleaved full-length cGAS and thus reduces the rate of enzyme activation and cyclic GMP-AMP synthesis. Furthermore, we found that NS2B3 physically interacts with full-length cGAS and CP-C, laying the basis for their shuttling to and eventual degradation in the autophagosome. Overall, our study highlights a multifaceted and effective strategy by which an RNA virus antagonizes cGAS-STING signaling which may be useful for the design of antivirals targeting viral proteases.

Dengue fever is one of the most widespread infectious diseases in the world. It afflicts 128 countries and more than one third of the world’s population is at the risk of infection (1). Dengue fever is caused by Dengue virus (DENV) which is transmitted by *Aedes* (*Ae.*) species mosquitoes, and humans are the primary reservoir of infection. DENV belongs to the Flaviviridae family that also includes West Nile virus, Zika virus, and Yellow Fever virus. The positive RNA genome of DENV translates into a polypeptide that harbors three structural proteins and seven nonstructural (NS) proteins (2, 3). Processing of the polypeptide into individual viral proteins requires both cellular and viral proteases. Nonstructural protein 3 (NS3) is the major DENV protease that cuts at multiple NS junctions. NS3 is a bipartite protein with the N-terminal 185 amino acids (aa) harboring protease activity, while the C-terminal ∼420 aa forming a helicase. NS3 protease domain (aa 1–185) is a serine protease that requires a cofactor contributed by NS2B to achieve full activity. Part of NS2B is transmembrane that anchors NS2B to the endoplasmic reticulum, while the cofactor region (aa 49–95) provides stability to NS3 protease domain and promotes its activity (4). The minimal protease thus is composed of the NS2B cofactor region and the N-terminal protease region of NS3.

Cyclic GMP-AMP synthase (cGAS) is a dsDNA sensor that synthesizes cyclic GMP-AMP (cGAMP) from GTP and ATP upon binding to dsDNA (5, 6). The C-terminal nucleotidyltransferase domain (NTase) is sufficient to synthesize cGAMP in the presence of dsDNA (7). The N-terminal disordered region (aa 1–160) has been shown to bind DNA and enhance cGAMP production (8, 9). cGAMP then binds to and activates endoplasmic reticulum–residing STING (6), leading to STING translocation, conformational change, and oligomerization (10). Activated STING then activates the kinase TBK1, which phosphorylates and activates the transcription factor interferon regulatory factor 3, initiating transcription of type I interferons and launching the cell into a potent antiviral state.

cGAS activation is normally elicited by DNA viruses such as herpes simplex virus 1 and Kaposi’s sarcoma-associated herpesvirus (11). However, recent studies uncovered that the cGAS-cGAMP-STING axis is also activated upon DENV infection (12, 13). cGAS activation upon DENV infection may be indirect, through mitochondria damage and leakage of mitochondrial DNA into the cytoplasm (12, 13). The importance of cGAS-STING activation in counteracting DENV infection is underlined by the fact that DENV actively suppresses both cGAS and STING (14, 15). DENV protease NS2B3 has been found to cleave and inactivate human STING but not murine STING (14). NS2B is also required for autophagosome-mediated cGAS degradation (12). However, whether NS2B3 directly cleaves cGAS as it does STING is unclear. Though the protease activity of NS2B3 was initially proposed to be essential for cGAS degradation, later it was shown that the NS2B cofactor alone is sufficient to shuttle cGAS to autophagosomes, while the function of the protease activity is to liberate the NS2B cofactor from the DENV polypeptide (14). As the NS2B3-mediated proteolytic cleavage of cGAS and NS2B-dependent autophagosomal degradation of cGAS may coexist in cells, it is difficult to distinguish contributions from the two events.

In work described in this article, we employed an *in vitro* recombination system to investigate whether NS2B3 cleaves cGAS without the interference from other types of degradation. We discovered that fully active NS2B3 proteolytically cleaves cGAS into two major fragments. Using site-directed mutagenesis and mass spectrometry, we mapped the major cleavage site to R124-G125 in the N-terminus of cGAS. We also identified a minor cleavage site between K62 and S63. Cleavage *per se* does not disrupt the C-terminal NTase domain. Rather, it elevates the threshold for cGAS activation by reducing the affinity to dsDNA. Furthermore, the cleft N-terminal fragment competes with the C-terminal NTase domain for dsDNA binding, inhibiting cGAMP production in *trans* by further reducing dsDNA availability. Lastly, the active NS2B3 protease interacts with both full-length (FL) cGAS and the cleavage product bearing the NTase domain, enabling their delivery to autophagosomes for further degradation. Collectively, our data clearly shows that the DENV NS2B3 protease is capable of cleaving cGAS and suppressing cGAMP production *in vitro*.

## Results

### NS2B3 cleaves human cGAS *in vitro*

**Figure 1:**
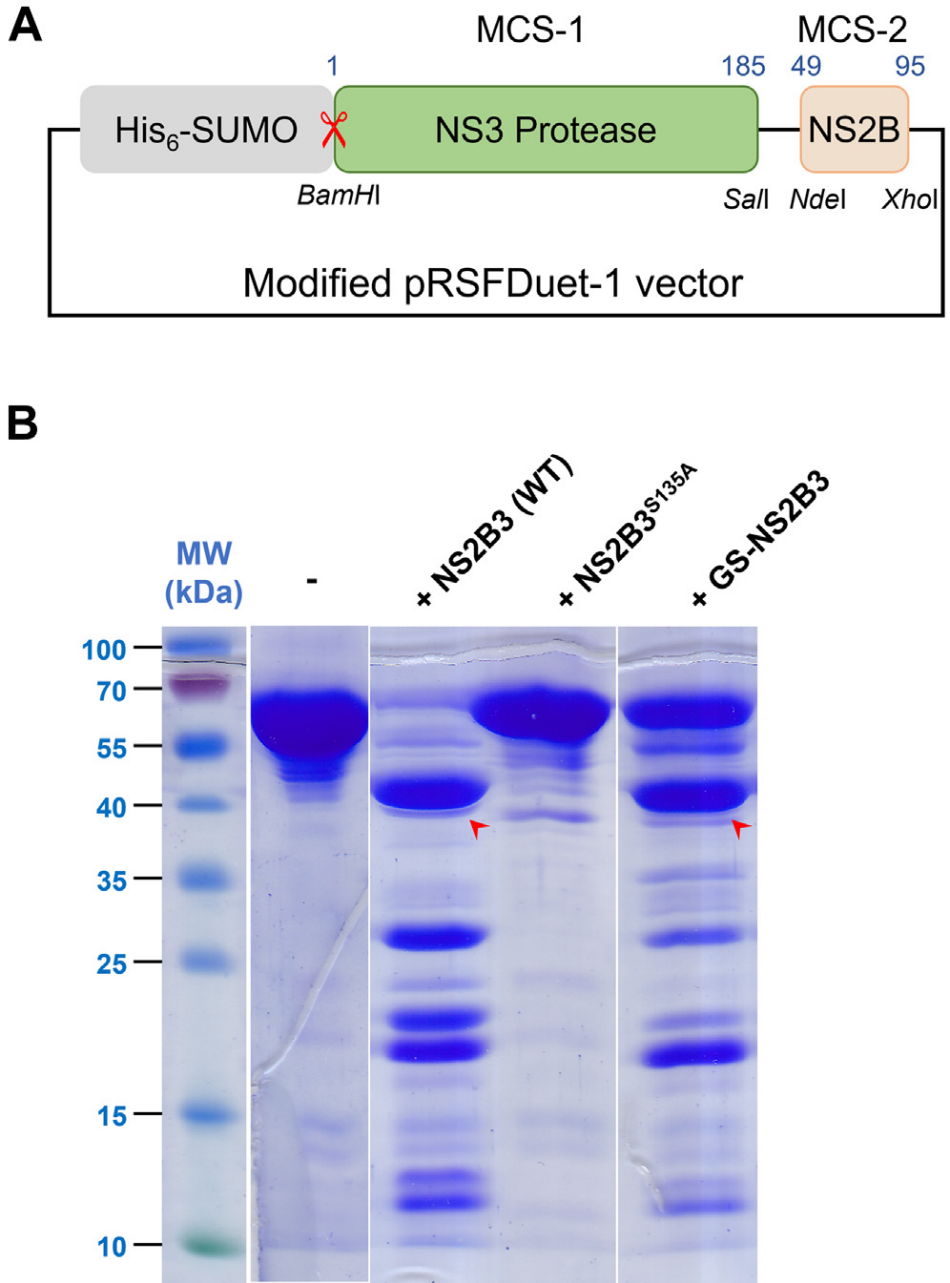
**DENV NS2B3 protease cleaves human cGAS in solution.***A*, a schematic diagram of coexpression of the NS2B hydrophilic core and the NS3 protease domain using a Duet vector. *B*, SDS-PAGE showing WT NS2B3, but not the active site mutant NS2B3^S135A^, cleaves purified recombinant cGAS. Covalently linked GS-NS2B3 cleaves cGAS with less efficiency. The *red arrowheads* point to the major cleavage product. cGAS, cyclic GMP-AMP synthase; DENV, Dengue virus; NS, nonstructural.

The DENV genome translates into a single polypeptide that requires both cellular and viral proteases for its processing into mature proteins. In the mature form of the protease NS2B3, the polypeptide is cleaved between NS2B and NS3. Many previous works on DENV NS2B3Pro utilize a covalently linked version in which the NS2B and NS3 are linked by a Gly4-Ser-Gly4 linker (G4SG4) which mimics the preprocessing form of NS2B3Pro (16) (Fig. S1*A*). However, more recent studies revealed that the covalent linker causes steric hindrance for substrate binding and hence affects the enzymatic efficiency of the Zika virus NS2B3 protease, a close relative of DENV NS2B3Pro (17, 18) (Fig. S1*B*). To preclude any possibility of interference with NS2B3 protease activity, we coexpressed the NS2B and NS3Pro genes in a Duet vector (Fig. 1*A*). After expression, the two polypeptides spontaneously folded together to form one entity as shown by the size-exclusion chromatography (SEC) profile and the SDS-PAGE gel (Fig. S1, *C* and *D*). We name this construct NS2B3 for the sake of brevity in this article. As a control, we also expressed and purified the covalently linked NS2B-G4SG4-NS3Pro version (GS-NS2B3 hereafter) as previously reported (Fig. S1, *C*, *E* and *F*).

We then examined whether NS2B3 is capable of cleaving cGAS *in vitro*. Recombinant FL human cGAS was expressed with an N-terminal His_6_ purification tag and purified as previously reported (19). cGAS was incubated with WT NS2B3, the active site mutant (NS2B3^S135A^), or the covalently linked GS-NS2B3. After 3 h, the mixtures were examined by SDS-PAGE (Fig. 1*B*). When incubated with WT NS2B3, the FL cGAS band completely disappeared while a new band with a molecular mass of ∼ 40 kDa appeared (Fig. 1*B*), suggesting the FL cGAS was processed into a major, stable fragment. In contrast, the FL cGAS band remained intact for the entire incubation period when incubated with the active site mutant NS2B3^S135A^ (Fig. 1*B*), confirming that the cleavage of FL cGAS is due to the enzymatic activity of NS2B3.

NS2B3 cleaves cGAS more efficiently than GS-NS2B3, echoing the Zika virus protease results (17, 18). In a time course experiment, cleavage of cGAS by NS2B3 could be detected as early as 5 min. At the end of the 3-h incubation time, more than 80% of cGAS was cleaved (Fig. S2, *A* and *C*). In contrast, cleavage of cGAS by GS-NS2B3 was not detected until 20 min. At the end of the incubation time, only less than half of cGAS was cleaved by GS-NS2B3 (Fig. S2, *B* and *C*).

### NS2B3 cleaves cGAS in the N-terminal region

**Figure 2:**
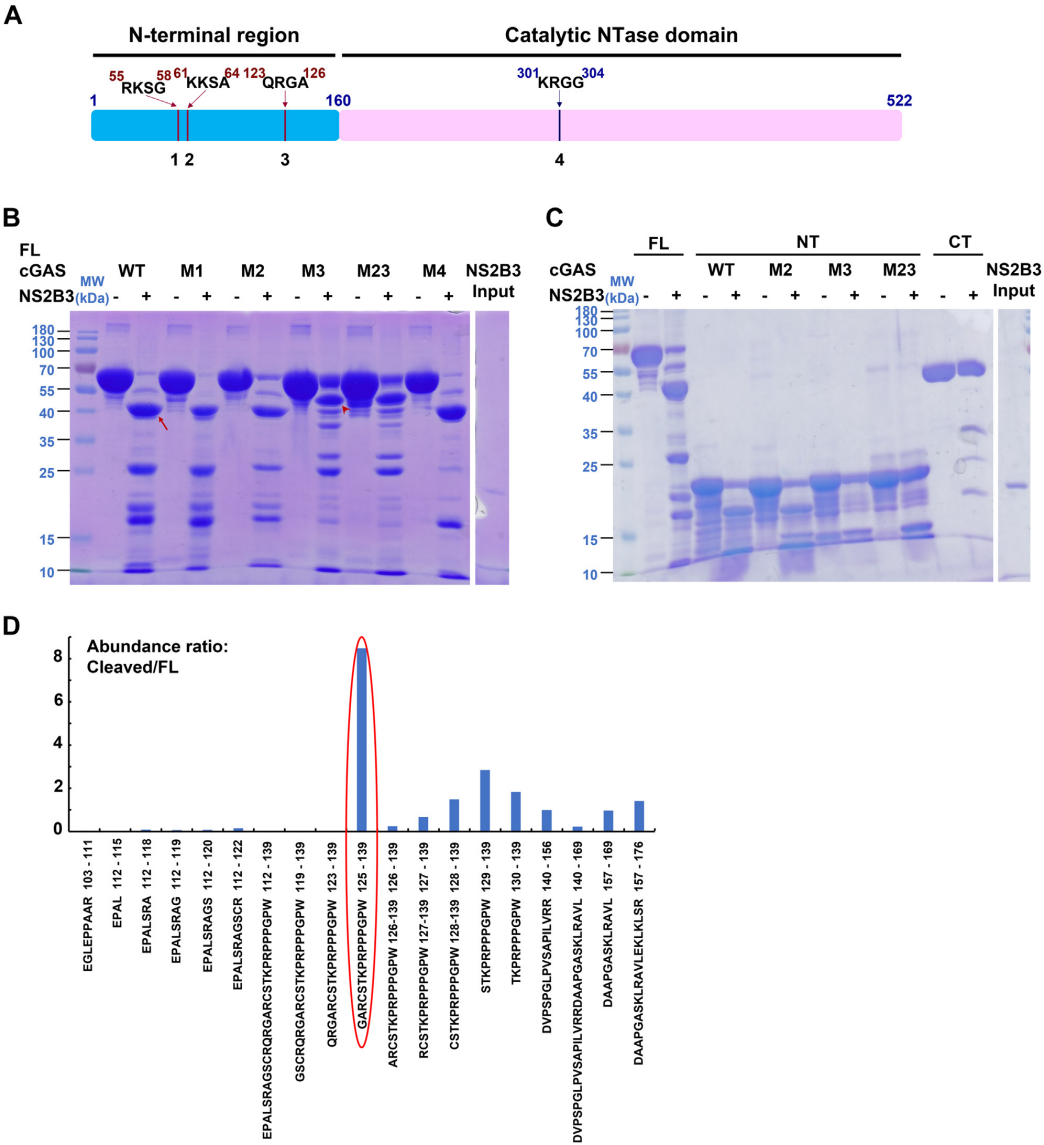
**Mapping NS2B3 cleavage sites to the N-terminal region of cGAS.***A*, a schematic diagram of cGAS with the two domains and four potential NS2B3 cleavage sites. *B*, SDS-PAGE of full-length (FL) WT and mutant cGAS (20 μM) incubated with NS2B3 (1 μM). The *red arrow* and *arrowhead* point to the major cleavage products in WT and M3 cGAS. *C*, SDS-PAGE of cGAS N-terminal region (NT) WT and mutants and WT C-terminal domain (CT) incubated with NS2B3. *D*, abundance ratio of peptides from cleaved *versus* FL cGAS after AspN digestion shown in the focused region of peptides 103 to 176. Minimal signal of cleaved protein was detected before amino acid 125, whereas comparable signals from cleaved and full-length cGAS were detected after amino acid 125. cGAS, cyclic GMP-AMP synthase; NS, nonstructural.

*In vitro* cleavage of cGAS by DENV NS2B3 generated a relatively stable intermediate of ∼40 kDa, suggesting a limited number of cleavage sites. We then sought to identify the cleavage site(s) in cGAS. NS2B3 protease strongly prefers a basic residue at the P1 site, an R, T, or Q/N/K at the P2 site, and small and polar residues at the P1′ site (20). cGAS contains four potential NS2B3 cleavage sites: three in the N-terminal region and one in the C-terminal NTase domain (Fig. 2*A*) (12). We mutated the four sites individually by replacing the charged/polar residues with alanines (M1: ^55^RKSG^58^ → ^55^AASG^58^; M2: ^61^KKSA^64^ → ^61^AASA^64^; M3: ^123^QRGA^126^ → ^123^AAGA^126^; and M4: ^301^KRGG^304^ → ^301^AAGG^304^). All four individual mutants were expressed and purified as the WT cGAS. When incubated with NS2B3 under same conditions, M1 and M4 displayed similar cleavage patterns as the WT cGAS (Fig. 2*B*), suggesting these two sites are not the targets of cleavage. M2 and M3, however, were more resistant to cleavage. M2 was processed to the same cleavage product but with less cleavage efficiency. Cleavage of M3 was even less efficient and produced an alternative fragment with an apparent molecular mass of ∼50 kDa (Fig. 2*B*). Cleavage at site 3 (after R124) or site 2 (after K62) will generate large protein fragments with molecular mass of 46.0 kDa and 52.6 kDa, respectively. The calculated protein fragment sizes are consistent with experimental band sizes. When both sites 2 and 3 were mutated, the double mutant M23 displayed a cleavage pattern similar to that of M3. A time-course cleavage assay comparing WT cGAS and M23 showed that at all time points examined, M23 was more resistant to NS2B3 cleavage than WT cGAS (Fig. S3). Together, these results suggest NS2B3 mainly cuts cGAS after residue R124 (site 3), but residue K62 (site 2) presents as an alternative cleavage site when site 3 is not available.

To further validate that NS2B3 cleaves cGAS in the N-terminal disordered region, we expressed and purified the individual domains of the N-terminal disordered region (NT, aa 1–156) and the C-terminal NTase domain (CT, aa 161–522). When incubated with NS2B3, cGAS NT was readily processed while the C-terminal NTase domain (CT) remained intact throughout the incubation period (Fig. 2*C*). When the same sites 2, 3, and 23 double mutations were introduced into the N-terminal region, site 2 mutation (NT_M2) displayed slight less cleavage than WT NT, while site 3 mutation (NT_M3) was more resistant to cleavage, consistent with the observations with FL cGAS. The sites 2 and 3 double mutant of cGAS NT (NT_M23) was even more resistant to NS2B3 cleavage, with more than half of cGAS remaining intact at the end of the incubation time (Fig. 2*C*). Together, the cleavage results with individual domains confirmed that R124 was the primary cleavage site by NS2B3 and K62 was a secondary cleavage site.

Independently, we sought to use mass spectrometry to define the primary cleavage site. FL cGAS and the ∼40 kDa major cleavage product bands were excised from the SDS-PAGE gel and digested by the endoproteinase AspN. Comparing the peptides generated from FL cGAS and the ∼40 kDa cleavage band demonstrated that the first identifiable peptide from the cleavage product band starts from G125 (Figs. 2*D* and S4; Table S1). The mass spectrometry results fully corroborate our conclusion that the primary cleavage site lies between R124 and G125 of human cGAS.

### Cleavage reduces cGAS binding to DNA and cGAMP production

**Figure 3:**
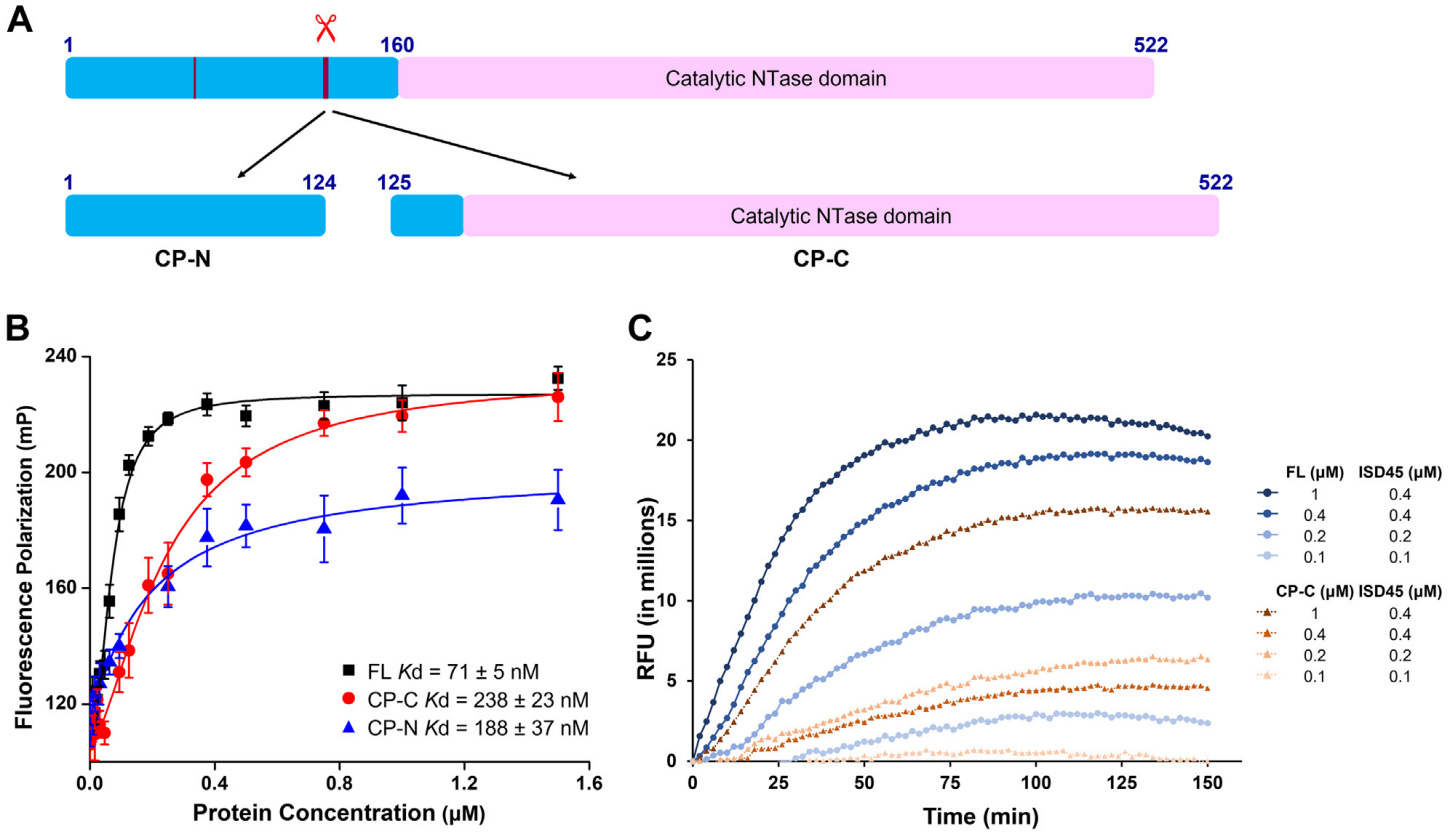
**Cleavage reduces cGAS affinity to DNA and cGAMP synthesis activity.***A*, schematic diagram showing the two cleavage products of cGAS (cleavage products CP-N and CP-C) after R124. *B*, DNA-binding affinities of full-length (FL) cGAS, CP-N, and CP-C measured by fluorescence polarization. The *binding curves* shown here are representative of two independent measurements. *C*, comparison of cGAMP synthesis activities of FL cGAS and the CP-C fragment at various concentrations. The *curves* shown here are representative of three independent experiments. cGAMP, cyclic GMP-AMP; cGAS, cyclic GMP-AMP synthase; CP-C, cleavage product C-terminal; CP-N, cleavage product N-terminal; RFU, relative fluorescence units.

NS2B3 cleavage after R124 does not compromise the structural integrity of the catalytic NTase domain, the domain that is responsible for DNA-induced cGAMP synthesis (Figs. 3*A* and S5). This raised the question that whether the cleavage affects cGAS activity at all, and if yes, to what degree and by what means. To investigate the effects of NS2B3 cleavage on cGAS, we constructed two cGAS fragments representing the primary cleavage products: cleavage product N-terminal (CP-N, 1–124) and cleavage product C-terminal (CP-C, 125–522). We then examined the DNA-binding affinities and cGAMP synthesis of the two cleavage products. Using fluorescence polarization (FP), we measured that FL cGAS bound to ISD45 (interferon stimulatory DNA, a 45-bp non-CpG oligomer from the *Listeria monocytogenes* genome) with a binding affinity of 71 ± 5 nM (Fig. 3*B* and Table S2), comparable to our previous results (19). CP-N bound to ISD45 with a slightly lower affinity of 188 ± 37 nM, a ∼2.5 fold reduction. CP-C bound to ISD45 with an even lower affinity of 238 ± 23 nM, showing a 3.3 fold reduction. We then employed a fluorescence-based assay to assess cGAMP synthesis by FL cGAS and CP-C. A fluorescent ATP analog 2-aminopurine riboside-5′-O-triphosphate (fATP) is used as the substrate. Once incorporated into cGAMP, the fluorescent signal decreases (Fig. S6) (15, 19, 21). Consistent with the lowered DNA-binding affinity, CP-C was less active than FL cGAS in synthesizing cGAMP at all concentrations (Fig. 3*C*). Interestingly, when we further examined the enzymatic activity of FL cGAS and CP-C by varying ATP concentrations (22), the *K*m values of FL cGAS and CP-C are comparable (Fig. S7 and Table S3), indicating the low cGAMP yield by CP-C is due to the lower DNA-binding affinity instead of compromised catalysis. These data collectively demonstrate that though NS2B3 cleavage does not directly disrupt the cGAS catalytic domain, the resultant cleavage product is less efficient in DNA binding and cGAMP production.

### The N-terminal fragment CP-N inhibits cGAS in *trans* by competing for DNA

**Figure 4:**
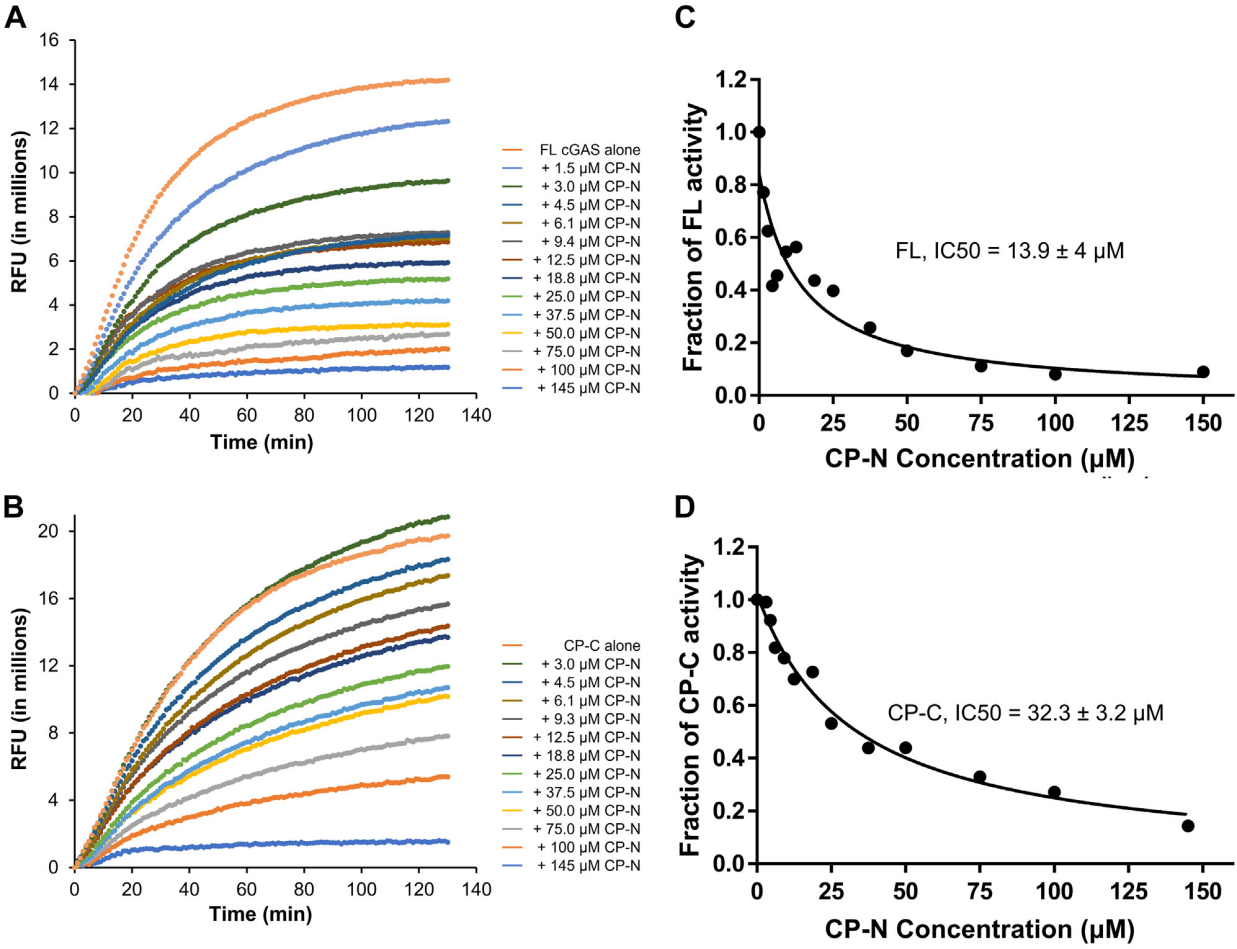
**CP-N inhibits cGAMP production by full-length cGAS and CP-C.***A* and *B*, time-course fluorescence showing full-length (FL) cGAS (*A*) and CP-C (*B*) activities in the presence of increasing concentrations of CP-N. *C* and *D*, plot of FL cGAS (*C*) or CP-C (*D*) activity in the presence of increasing CP-N concentrations. Results shown here are representative of at least three independent experiments. cGAMP, cyclic GMP-AMP; cGAS, cyclic GMP-AMP synthase; CP-C, cleavage product C-terminal; CP-N, cleavage product N-terminal; RFU, relative fluorescence units.

The N-terminal disordered region of cGAS (1–156), while incapable of cGAMP synthesis, binds to DNA and promotes FL cGAS binding to DNA and cGAMP synthesis activity (8, 9). NS2B3 cleavage of cGAS produced an N-terminal fragment CP-N (1–124), encompassing 75% of the N-terminal disordered region. The isoelectric point (pI) of CP-N is 11.25, strongly suggesting its capability to bind DNA as well. Indeed, we observed that CP-N binds to ISD45 with an affinity of 188 nM, less than FL cGAS, but comparable to the CP-C region (Fig. 3*B*). Since CP-N itself does not possess any cGAMP synthesis activity (Fig. S8), we postulate that CP-N can compete with FL cGAS or CP-C for any available DNA and further reduces cGAMP production. Using the same fluorescence-based cGAS activity assay, we found that inclusion of CP-N indeed reduced cGAS-dependent cGAMP production in a dose-dependent manner (Fig. 4*A*). CP-N inhibited cGAMP production by FL cGAS with an estimated IC50 value of 13.9 ± 4 μM when cGAS concentration was fixed at 1 μM (Fig. 4*C* and Table S4). CP-N also inhibited CP-C activity with an IC50 value of 32.3 ± 3.2 μM (Fig. 4, *B* and *D* and Table S5). We conclude that though not directly involved in cGAMP production, the presence of CP-N further reduces the ability of FL cGAS or CP-C to synthesize cGAMP.

### NS2B3 interacts with both FL cGAS and CP-C

**Figure 5:**
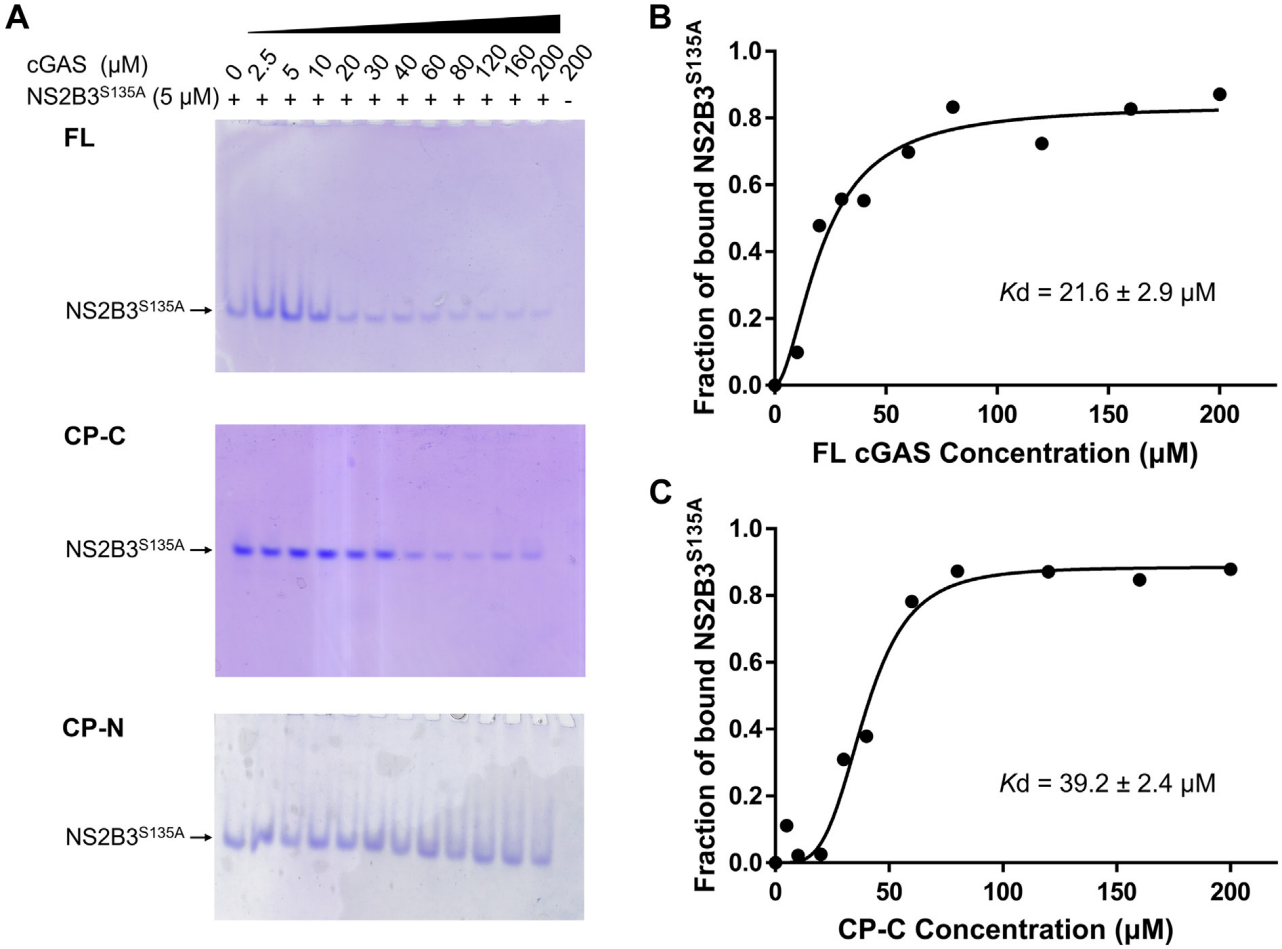
**NS2B3 interacts with full-length cGAS and the CP-C fragment.***A*, gel shift assay showing NS2B3^S135A^ interacts with full-length (FL) cGAS, CP-C, but not CP-N. Concentrations of each protein are marked on *top* of the gels. *B* and *C*, plots of bound NS2B3^S135A^ fractions in the presence of increasing concentrations of FL cGAS (*B*) or CP-C (*C*) to derive binding affinities. Results shown here are representative of two independent measurements. cGAS, cyclic GMP-AMP synthase; CP-C, cleavage product C-terminal; CP-N, cleavage product N-terminal; NS, nonstructural.

It is reported that NS2B3, even in its protease-inactive form, mediates cGAS degradation by shuttling cGAS to autophagosomes (12). However, it is unclear whether the interaction between NS2B3 and cGAS is direct or requires other factors. To characterize the interaction, we carried out native PAGE between the two proteins. We adopted the active site mutant NS2B3^S135A^ to preclude any accidental cleavage of cGAS or CP-C. With a pI of 9.54, cGAS does not enter the gel. The interaction is therefore monitored as decreases in NS2B3 band intensities. We observed that NS2B3 band intensity steadily diminished in the presence of increasing amount of FL cGAS (Fig. 5*A*). The binding affinity was estimated to be 21.6 ± 2.9 μM (Fig. 5*B* and Table S6). NS2B3 similarly interacts with CP-C with an estimated affinity of 39.2 ± 2.4 μM (Fig. 5, *A* and *C* and Table S6). We did not detect any interaction between NS2B3 and CP-N (Fig. 5*A*), echoing previously published results (12).

## Discussion

**Figure 6:**
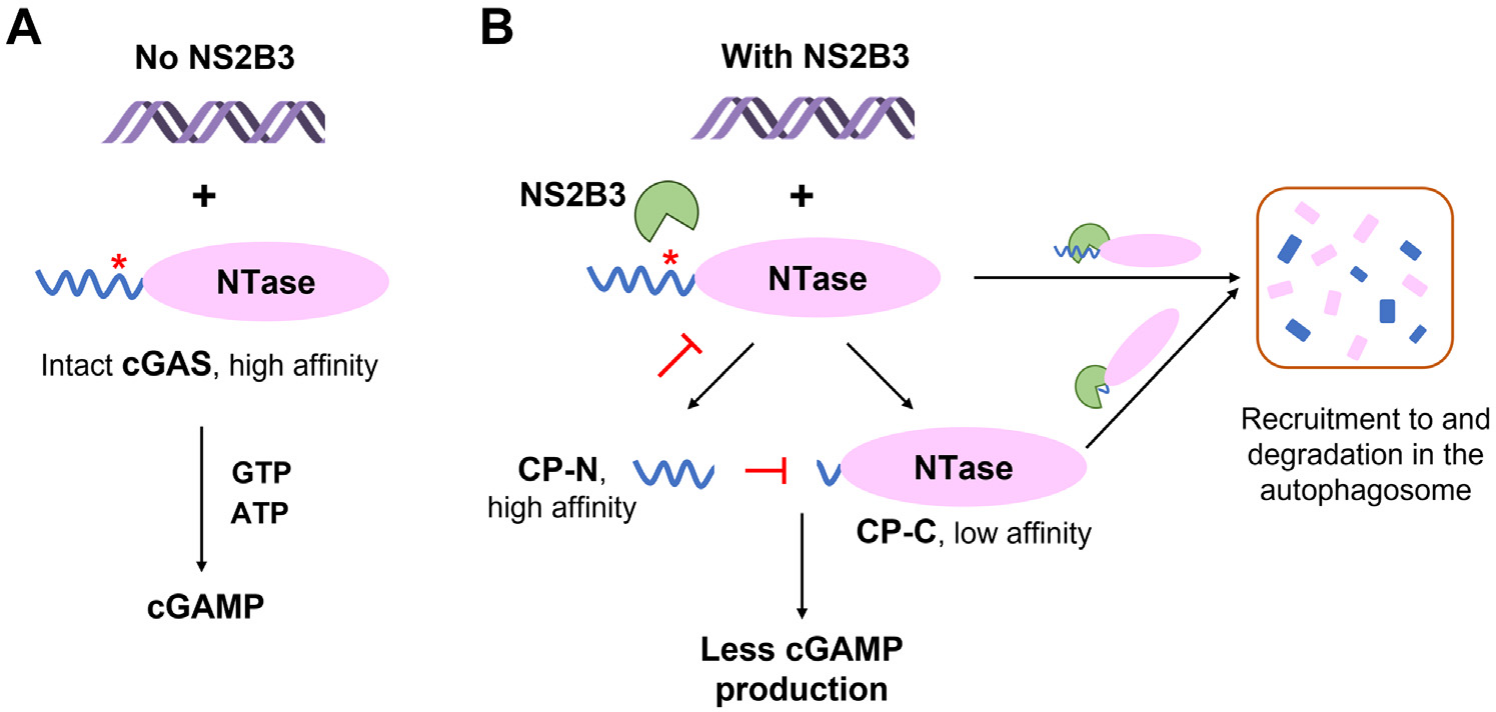
**Diagram of multifaceted inhibitory effects of DENV NS2B3 on cGAS.***A*, in the absence of NS2B3, cGAS detects dsDNA and synthesizes cGAMP from GTP and ATP. *B*, in the presence of NS2B3, cGAS is cleaved into the CP-N and CP-C fragments, the latter of which is less active than full-length cGAS; CP-N sequesters dsDNA from full-length cGAS and CP-C, further reducing cGAMP production; independent of proteolytic cleavage, NS2B3 shuttles full-length cGAS and CP-C to the autophagosome for degradation. ∗ the major cleavage site in the N-terminus of cGAS. cGAMP, cyclic GMP-AMP; cGAS, cyclic GMP-AMP synthase; CP-C, cleavage product C-terminal; CP-N, cleavage product N-terminal; DENV, Dengue virus; NS, nonstructural.

In this study, we discovered that the DENV protease NS2B3 is capable of cutting human cGAS after residue R124 *in vitro*, and to a minor degree, after K62 (Fig. 2). Additional cleavage sites in the N-terminal region of cGAS are also observed when R124 and K62 sites are mutated (Figs. 2 and S3), likely due to the disordered nature of the N-terminal region. Though the cleavage does not disrupt the catalytic NTase domain, the resultant C-terminal fragment (CP-C, 125–522) binds to DNA with decreased affinity, reducing its responsiveness to the presence of aberrant DNA. Furthermore, the N-terminal fragment (CP-N, 1–124) binds to DNA with moderate affinity, making it a competitor for any available DNA that may activate intact cGAS or CP-C. Lastly, NS2B3 physically interacts with both FL cGAS and CP-C, likely lay the physical basis for shuttling to autophagosome for degradation (Fig. 6). Further cell-based experiments will reveal whether NS2B3 cleavage of cGAS holds true in cells and constitutes part of the DENV strategy to suppress host antiviral immunity.

Some of our findings appear inconsistent with previous publications, *e.g.*, NS2B3^S135A^ did not interact with cGAS (12). As the previous work relied on self-cleavage of the NS2B3 peptide, the active site mutation S135A resulted in covalently linked NS2B and NS3 peptides (12). Both our data and another publication on Zika virus NS2B3 have shown the covalent link between the two peptides negatively impact the protease activity of the enzyme (Figs. 1*B* and S2) (17, 18), likely contributing to the discrepancies in our results and the previous publication.

Inhibition of host innate immune machinery is a common strategy for viruses to establish infection. As cGAS-cGAMP-STING axis is the major DNA-sensing pathway for interferon production, DNA viruses are known to evade or actively suppress the activation of cGAS or STING (23). However, RNA viruses are also reported to suppress the cGAS-cGAMP-STING pathway. Aside from DENV, ZIKV, and Chikungunya virus, Yellow fever virus, hepatitis C virus, and SARS also encode viral proteins to interfere with varying aspects of cGAS-cGAMP-STING signaling (24, 25). DENV protease NS2B3 has been found to cleave and inactivate human STING but not murine STING (14). It remains to be seen whether cGAS manifests similar species-dependent susceptibility to NS2B3 cleavage. The knowledge will inform the development of mouse model of DENV infection.

R124 resides in an R/K-rich region that binds to DNA *in vitro* and is essential to full cGAS activation in cells (8, 26). Cleavage after R124 compromises the integrity of this region, resulting in diminished DNA binding and cGAMP production (Fig. 3, *B* and *C*). Cleavage in the N-terminus region may affect cellular homeostasis in addition to antiviral responses. The N-terminus of cGAS binds to PtdIns(4,5) P_2_ and anchors cGAS to the inner leaflet of plasma membrane (27). Mislocalization of cGAS to cytosolic or nuclear compartments induces genotoxic stress but weaker responses to viral infection (27). Cleavage after R124 by DENV NS2B3 frees the catalytic NTase domain from the plasma membrane. Mislocalization of the NTase domain will likely lead to dysregulated cell responses in addition to reduced antiviral responses.

Cellular proteases have been reported to cleave cGAS and dampen the interferon responses as well. The inflammatory caspase-1 cleaves cGAS in the N-terminal region, after D140 and D157, essentially separating the N-terminal region from the C-terminal catalytic domain (26). Such cleavage impairs cellular cGAMP production and interferon responses. Pyroptotic caspases caspase-4, caspase-5, and caspase-11 also cleave cGAS in the N-terminal region, though with different cleavage sites. Interestingly, ZIKV NS protein NS1 stabilizes caspase-1 to promote cGAS cleavage and impair interferon responses (28). The fact that both viral and cellular proteases target the N-terminal region of cGAS highlights the importance of the N-terminal region in cGAS activation and suggests a converging theme of targeting the N-terminal region for cGAS downregulation.

## Experimental procedures

### Cloning and mutagenesis

FL human cGAS and the CT constructs were from previous studies (19, 29). Other human cGAS constructs (CP-C, CP-N, and NT) were generated by standard PCR protocol using the designed primers (Integrated DNA Technologies) and inserted into to a modified pET28a vector (Novagen) between the *Nde*I and *Sal*I sites. The resultant polypeptides bear a 3C protease site between the His_6_ tag and the cGAS sequence. The NS2B3 coexpression construct was created by inserting the sequences encoding NS3 protease domain (1–185) and the hydrophilic core of NS2B (49–95) into the multiple cloning sites 1 and 2 of a modified pRSFDuet-1 vector bearing an N-terminal His_6_-SUMO fusion tag (30). The covalently linked NS2B-G4SG4-NS3Pro construct was generated by overlapping PCR and insertion into the multiple cloning site 1 of the modified pRSFDuet-1 vector. All mutant constructs were generated using the designed mutagenesis primers and the QuikChange Site-Directed Mutagenesis kit (Agilent) following the manufacturer’s instruction.

### Protein expression and purification

All proteins were overexpressed in *Escherichia coli* BL21(DE3) RIPL cells (Agilent). Cells were grown in LB medium containing 50 mg/ml kanamycin and 34 mg/ml chloramphenicol at 37 °C. The culture was induced with 0.4 mM IPTG when A600 reached 0.6 and grown for 16 h at 20 °C. Cells were harvested by centrifugation at 4000*g* for 20 min. Cell pellets were resuspended in lysis buffer (50 mM sodium phosphate pH 7.4, 300 mM NaCl, 20 mM imidazole, 5 mM β-mercaptoethanol, and 10% glycerol) and lysed by sonication. Cell lysates were centrifugated at 15,000*g* for 45 min to remove cell debris. The supernatant was loaded onto a Ni-NTA gravity column equilibrated with the lysis buffer. The columns were then extensively washed with wash buffer (50 mM sodium phosphate pH 7.4, 300 mM NaCl, 30 mM imidazole, and 5 mM β-mercaptoethanol). NS2B3 proteins were then eluted in the elution buffer (50 mM sodium phosphate pH 7.4, 300 mM NaCl, 300 mM imidazole, and 5 mM β-mercaptoethanol). For NS2B3 proteins, the eluates were incubated with the protease Ulp1 (w:w 1:1000) at 4 °C overnight to cleave off the N-terminal His_6_-SUMO tag. The His_6_-SUMO tag was removed by a second round of Ni affinity chromatography. Tag-free NS2B3 proteins were further purified by SEC using a HiLoad 16/600 column (Cytiva) in a running buffer containing 20 mM Tris·HCl pH 8.0 and 150 mM NaCl. For all the cGAS constructs, the protein-loaded Ni-NTA column was first washed with wash buffer supplemented with 1 M NaCl, then with regular wash buffer. Bound proteins were eluted with elution buffer. cGAS elutes were then purified by heparin column chromatography with a 0 to 100% linear gradient from buffer A (20 mM Tris∙HCl pH 8.0, 300 mM NaCl) to buffer B (20 mM Tris∙HCl pH 8.0, 1 M NaCl). Peak fractions containing target proteins were further purified by SEC using a HiLoad 16/600 column (Cytiva) preequilibrated with running buffer (20 mM Tris∙HCl pH 7.4, 300 mM NaCl, and 5% glycerol). Peak fractions from SEC were concentrated, aliquoted, flash frozen in liquid nitrogen, and stored at −80 °C.

### Cleavage assay

20 μM FL cGAS, cGAS fragments, or cGAS mutants was mixed with 1 μM WT NS2B3, NS2B3^S135A^ mutant, or 1 μM GS-NS2B3 in a reaction volume of 10 μl and incubated at 37 °C for 3 h. For the time-course experiment, the reaction volume was 100 μl. Ten microliter aliquots were taken at indicated time points (0, 5, 10, 20, 30, 45, 60, 120, and 180 min). The cleavage reaction is terminated by the addition of SDS-PAGE loading dye into the reaction mixture. The reaction mixtures were analyzed by SDS-PAGE.

### In-gel protein AspN digestion

Cut gel bands were destained with a wash buffer (50% acetonitrile in H_2_O with 100 mM ammonium bicarbonate ABC). Destained gel bands were cut into ∼1 mm pieces, dried, and rehydrated with a digestion buffer (10% acetonitrile in H_2_O with 50 mM ABC). Protein was reduced with DTT and alkylated with iodoacetamide. After the reductive alkylation, the gel pieces were dried and rehydrated with the digestion buffer. Endoproteinase AspN (Thermo Fisher Scientific, Catalog # PI90053) was added and the mixture was incubated at 37 °C overnight. Digestion was quenched with the addition of a 0.5% formic acid aqueous solution and the supernatant was collected. The gel pieces were dried by incubation with acetonitrile and the supernatant was collected. Combined supernatant was dried in a SpeedVac (Thermo Fisher Scientific).

### Nano-liquid chromatography tandem mass spectrometry

The peptide mixture was redissolved in 5% acetonitrile in H_2_O with 0.1% formic acid. Peptides were separated by nLC with an Easy nLC-1200I system (Thermo Fisher Scientific) equipped with a nanoViper Acclaim PepMap 100 C18 100 μm × 2 cm trap column (Thermo Fisher Scientific, catalog # 164564-CMD) and a nanoViper Acclaim PepMap RSLC C18 75 μm × 15 cm analytical column (Thermo Fisher Scientific, catalog # 164534). Mobile phases are A (99.9% H_2_O with 0.1% formic acid) and B (90% acetonitrile with 0.1% formic acid). A stepwise linear gradient profile is as followed: from 0% to 1% B over 2 min, from 1% to 55% B over 1 h, from 55% to 85% B over 5 min, hold at 85% B for 10 min, from 85% to 1% B over 1 min, and hold at 1% B for 8 min with a flow rate of 300 nl/min. Eluate was on-line ionized with a nano-spray ion source at 2.3 kV. Ionized peptides were detected by a high mass accuracy high resolving power Orbitrap Exploris 480 Mass Spectrometer (Thermo Fisher Scientific). The precursor ions were detected with a mass resolution of 120 K (at m/z of 200 Da) with a mass range of 300 to 1500 in the Ultra-High-Field Orbitrap Mass Analyzer in the positive ion mode. Data-dependent MS2 on the top 15 most abundant precursor ions with higher-energy collisional dissociation were detected with a mass resolution of 15 K (at m/z of 200 Da) with a dynamic exclusion duration of 30 s and 10 ppm mass tolerance.

### Mass spectrometry data analysis

Mass spectrometry data was collected with the Xcalibur acquisition software (Thermo Fisher Scientific). Thus, generated .raw files were then analyzed with the Proteome Discoverer 2.5 software with Sequest HT (Thermo Fisher Scientific) against the cGAS sequence with dynamic modifications of methionine oxidation (+15.995 Da) and cysteine carbamidomethylation (+57.021 Da) and N-terminus methionine-loss and/or N-terminus acetylation. Identified peptide sequences were validated with a 1% strict target FDR (False Discovery Rate) and a 5% relax target FDR. To figure out the cleavage site, a typical workflow for label-free quantification (31) was adopted to compare the abundance of peptides of the cleaved *versus* FL cGAS (32). Peptide abundance data was exported to excel sheet for further analysis to plot the abundance ratio along the progression of protein sequence to find the peptide with the sudden “jump” in ratio.

### DNA-binding assay by FP

DNA binding by cGAS and cGAS fragments CP-C and CP-N was measured by FP. FP values were measured using 384-well black nonbinding PS plates (Greiner Bio-One) in a multimode micro-plate reader from Biotek Instruments. Increasing concentrations of FL cGAS, CP-C, or CP-N were added to a fixed concentration (12.5 nM) of FAM-labeled double stranded ISD45. FP values were plotted as a function of protein concentration and fitted to the Hill equation using Origin 8.5.

### Fluorescence-based cGAS activity assay

A fluorescent analog of ATP, 2-aminopurine riboside-5′-O-triphosphate (fATP) (Jena Biosciences) was used to assay cGAS activity (15, 19). ISD45 of indicated concentration was premixed with equimolar of cGAS proteins in a reaction buffer of 40 mM Tris·HCl pH 7.4, 100 mM·NaCl, and 600 μM of Zn^2+^. The reaction was started by the addition of 5 μM·MgCl_2_, 40 μM fATP, and 400 μM GTP. The real time decrease in fluorescence was measured in 384-well black nonbinding PS plates (Greiner Bio-One/Corning) at 37 °C on SpectraMax iD5 (λex = 307 nm, λem = 370 nm, gain 100, kinetic interval 1 min) for 150 min. The background fluorescence was subtracted from the initial fluorescence curve and the resulting curves were inverted for better visualization (15).

To determine the Michaelis-Menten parameters for cGAS and CP-C, each construct was incubated with ISD45 in the same manner as mentioned above. The substrate dependence of each construct was assessed by keeping the concentration of GTP fixed (1 mM) over a range of fATP concentrations (15–500 μM, 2-fold dilution) (22). The reactions were initiated by adding the mastermix containing ATP, GTP, and MgCl_2_ and monitored as mentioned above. The initial rates obtained for each reaction were plotted against ATP concentrations and the apparent *K*m values were calculated using the Michaelis-Menten module under Enzyme kinetics-substrate velocity in GraphPad Prism 7.

To examine the effect of CP-N on the activity of FL cGAS and CP-C, CP-N of indicated concentration was added to the mixture of ISD45 and cGAS (or CP-C), and the reaction was initiated and monitored as mentioned above. Activity is defined as the initial velocity V_0_ by extracting the slope of the initial linear phase. The fraction of FL cGAS activity is calculated as V_0_ (at a particular CP-N concentration)/V_0_ (FL cGAS or CP-C alone). The fractions are plotted against CP-N concentrations and fitted into the “Inhibitor *versus* Response” mode under “Dose Response-Inhibition” in GraphPad Prism 7.

### Native PAGE gel shift assay

Five micromolar NS2B3^S135A^ was incubated with increasing concentrations of cGAS or cGAS fragments for 3 h on ice in a buffer containing 20 mM Tris·HCl pH 7.4 and 100 mM NaCl. The samples were then examined by native PAGE. The band intensities were quantified using the ImageJ software (33) and normalized to the intensity of NS2B3^S135A^ alone. Fraction of free NS2B3^S135A^ were plotted against concentrations of cGAS or cGAS fragments and fitted to the equation “Receptor binding – Saturation binding: Specific binding with Hill slope” using GraphPad Prism 7.

## Data availability

All data are contained in this article.

## Supporting information

This article contains supporting information.

## Conflict of interest

The authors declare that they have no conflicts of interest with the contents of this article.
