# Supplementary material for: The Dengue virus protease NS2B3 cleaves cyclic GMP-AMP synthase to suppress cGAS activation
Source: J Biol Chem. 2023 Feb 7;299(3):102986. doi: 10.1016/j.jbc.2023.102986 (PMC10011430; doi:10.1016/j.jbc.2023.102986)
Supplement: Supporting Figures S1–S8 [file mmc1.pdf]

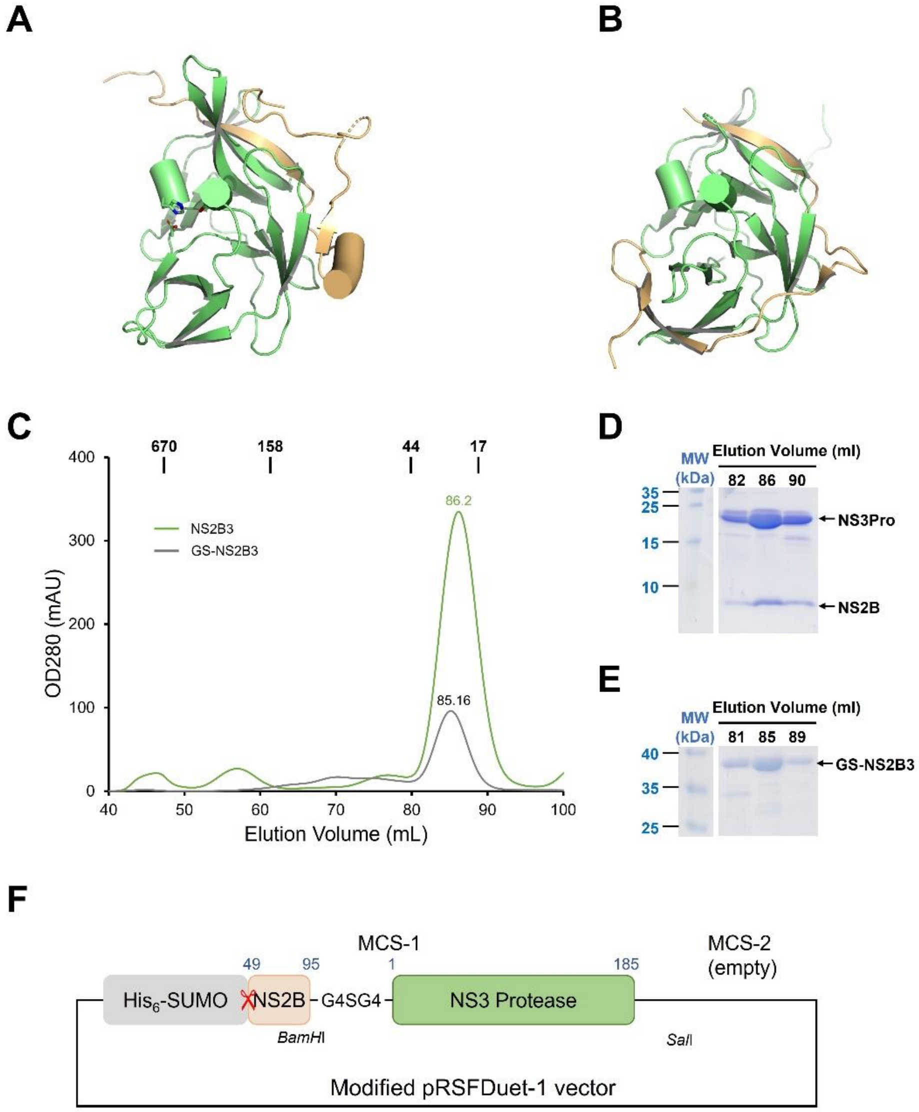

Figure S1 **Characterization of co-expressed and covalently linked NS2B3**. (*A*) Crystal structure of covalently linked DENV2 NS2B3 protease (GS-NS2B3, PDB ID: 2FOM). The NS2B and NS3Pro portions are colored in wheat and green, respectively. The catalytic triad is shown as sticks. (*B*) Crystal structure of co-expressed Zika virus NS2B3 protease (PDB ID: 5GPI). The structure is shown in the same orientation as DENV NS2B3 in (*A*). NS2B: wheat; NS3Pro: green. (*C*) Size exclusion chromatography profiles of NS2B3 (green trace) and GS-NS2B3 (gray trace). Elution positions of molecular standards are marked on the profiles. (*D-E*) SDS-PAGE of co-expressed NS2B3 (*D*) and GS-NS2B3 (*E*) eluted from the peak fractions in (*C*). (*F*) Schematic diagram of GS-NS2B3 expression construct. Gene fragments encoding NS2B (49-95) and NS3 protease domain (1-185) are linked by DNA encoding Gly4SerGly4 (G4SG4) and inserted into MCS1 in the modified pRSFDuet-1 vector.

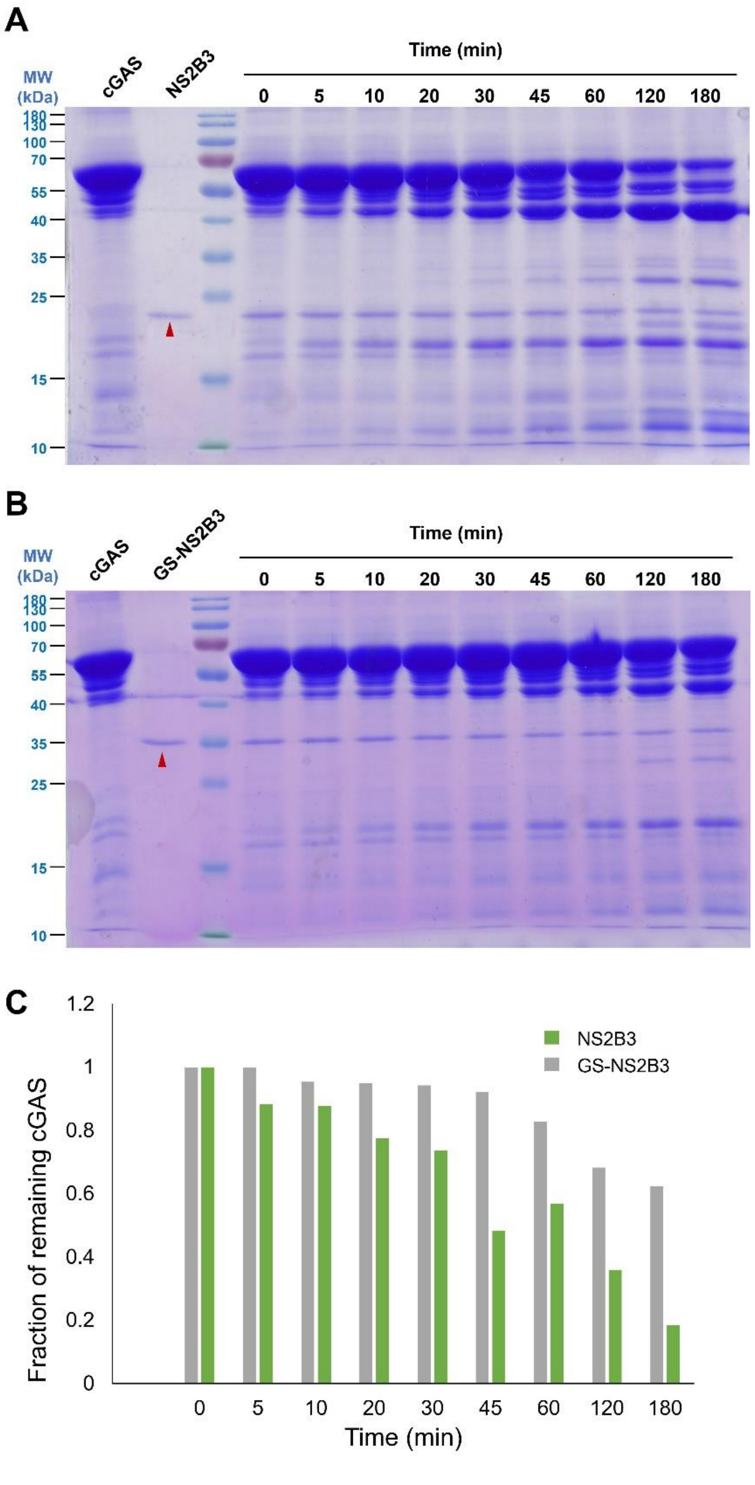

Figure S2 **Co-expressed NS2B3 is more active than the covalently linked GS-NS2B3**. (*A*-*B*). SDS-PAGE of cGAS cleavage patterns by NS2B3 (*A*) and GS-NS2B3 (*B*) at indicated time points. (*C*). Quantification of cGAS cleavage by NS2B3 (green trace) and GS-NS2B3 (gray trace) in (*A*-*B*).

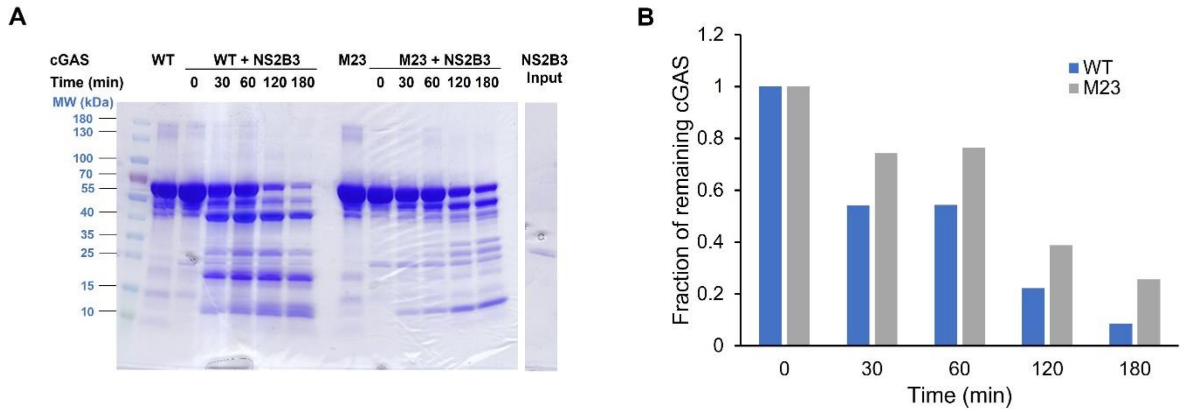

Figure S3 **Time course cleavage of wild-type (WT) cGAS vs the M23 mutant by NS2B3**. (*A*) SDS-PAGE gel resolving WT cGAS or M23 cleavage by NS2B3 at 0, 30, 60, 120, and 180 min. (*B*) Quantification of remaining cGAS in (*A*). Blue: WT cGAS; Gray: M23.

A

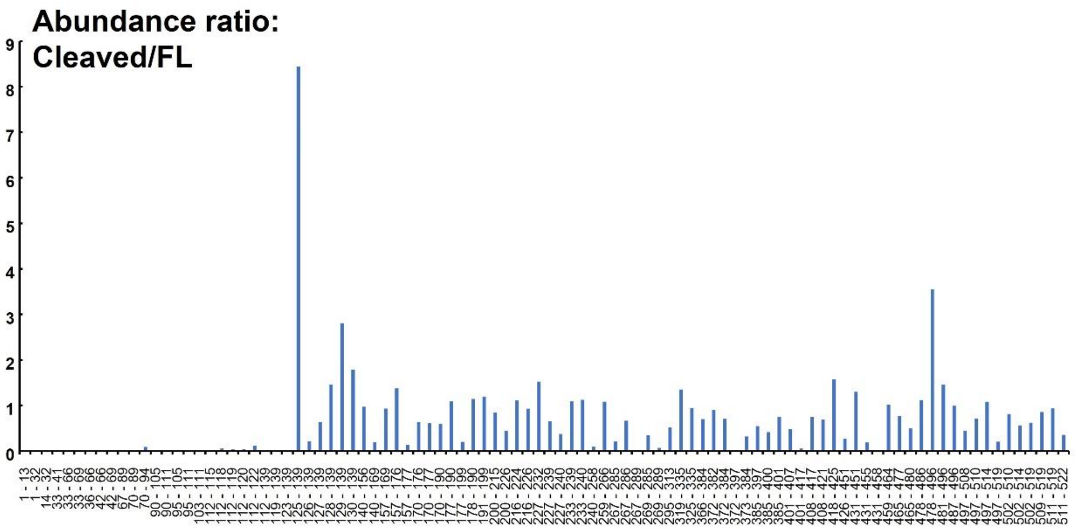

B

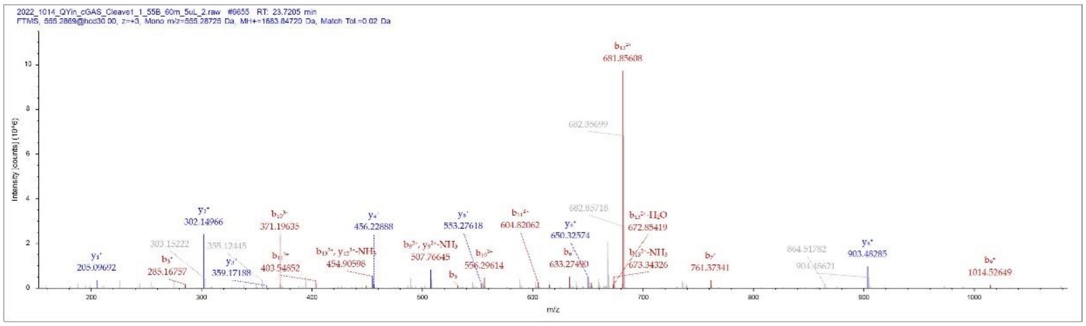

#1	b⁺	b^{2+}	b^{3+}	Seq.	y⁺	y^{2+}	y^{3+}	#2
1	58.02874	29.51801	20.01443	G				15
2	129.06585	65.03657	43.69347	A	1606.82709	803.91718	536.28055	14
3	285.16697	143.08712	95.72717	R	1535.78998	768.39863	512.60151	13
4	445.19761	223.10245	149.07072	Carbamidometl	1379.68887	690.34807	460.56781	12
5	532.22964	266.61846	178.08140	S	1219.65822	610.33275	407.22426	11
6	633.27732	317.14230	211.76396	T	1132.62619	566.81673	378.21358	10
7	761.37228	381.18978	254.46228	K	1031.57851	516.29289	344.53102	9
8	858.42505	429.71616	286.81320	P	903.48355	452.24541	301.83270	8
9	1014.52616	507.76672	338.84690	R	806.43078	403.71903	269.48178	7
10	1111.57892	556.29310	371.19783	P	650.32967	325.66847	217.44808	6
11	1208.63169	604.81948	403.54875	P	553.27691	277.14209	185.09715	5
12	1305.68445	653.34586	435.89967	P	456.22415	228.61571	152.74623	4
13	1362.70591	681.85659	454.90682	G	359.17138	180.08933	120.39531	3
14	1459.75868	730.38298	487.25774	P	302.14992	151.57860	101.38816	2
15				W	205.09715	103.05222	69.03724	1

Figure S4 **Mass spectrometry analyses of full-length cGAS and the cleavage product**. (*A*) Abundance ratio of peptides from cleaved vs. full-length cGAS after AspN digestion shown for the entire protein sequence. The horizontal axis shows the amino acid numbers of detected peptides. Minimal signal of cleaved protein was detected before amino acid 125 whereas comparable signals from cleaved and full cGAS were detected after amino acid 125. (*B*) Tandem mass spectrometry confirmed the identification of peptide GARCSTKPRPPPGPW (125-139).

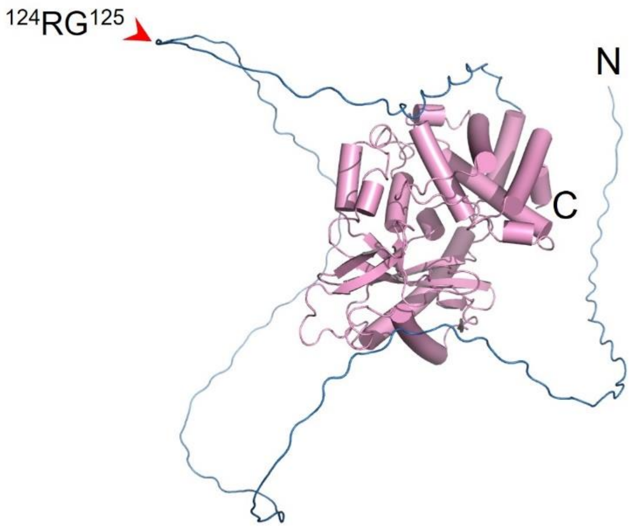

Figure S5 **The AlphaFold model of full-length cGAS**. The N-terminal region is colored in blue and the C-terminal NTase domain in pink. The N- and C-termini of cGAS and the major DENV NS2B3 cleavage site are marked on the structural model.

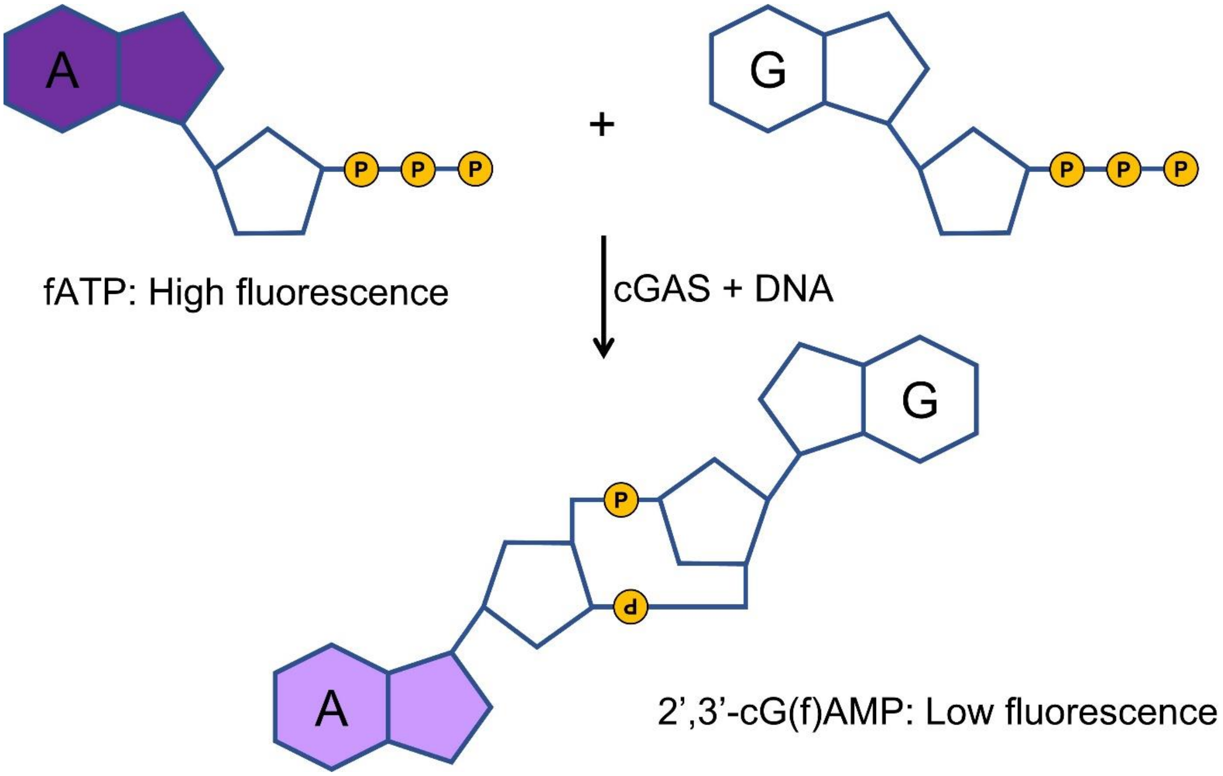

Figure S6 **Schematic diagram of fluorescence-based cGAS activity assay**. A fluorescent analog of ATP (2-aminopurine riboside-5′-O-triphosphate, fATP, $\lambda ex = 307$ nm, $\lambda em = 370$ nm) is used in the assay. cGAS converts fATP and GTP into 2',3'-cGAMP, which has lower fluorescence. Changes in fluorescence therefore can be used to assess cGAS activity.

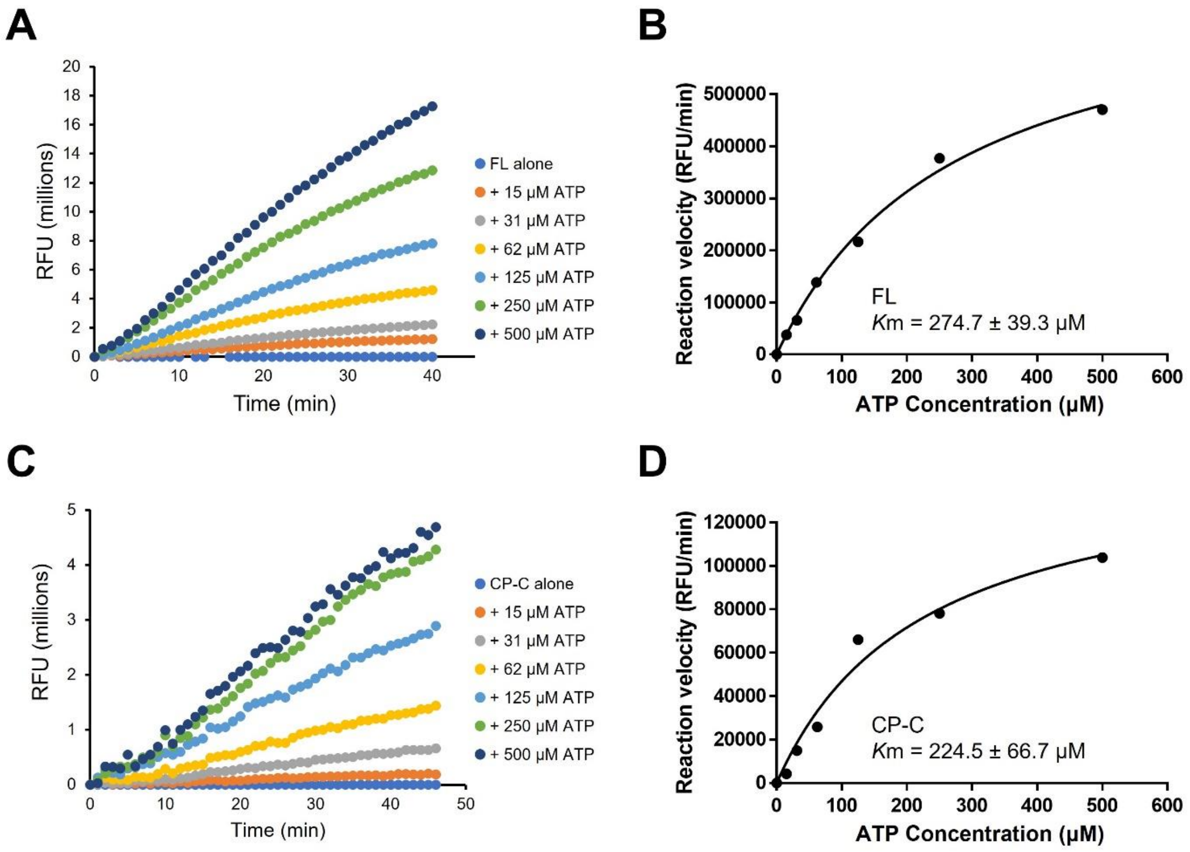

Figure S7 **Comparison of FL cGAS and CP-C enzymatic activity**. (*A*, *C*) FL cGAS (*A*) and CP-C (*C*) catalyze cGAMP synthesis at various fATP concentrations as shown by the changes in fATP fluorescence. (*B*, *D*) *K*m values of FL cGAS (*B*) or CP-C (*D*) derived from panels (*A*) and (*C*). The fluorescence time-course curves and *K*m calculation shown here are representative of three independent measurements.

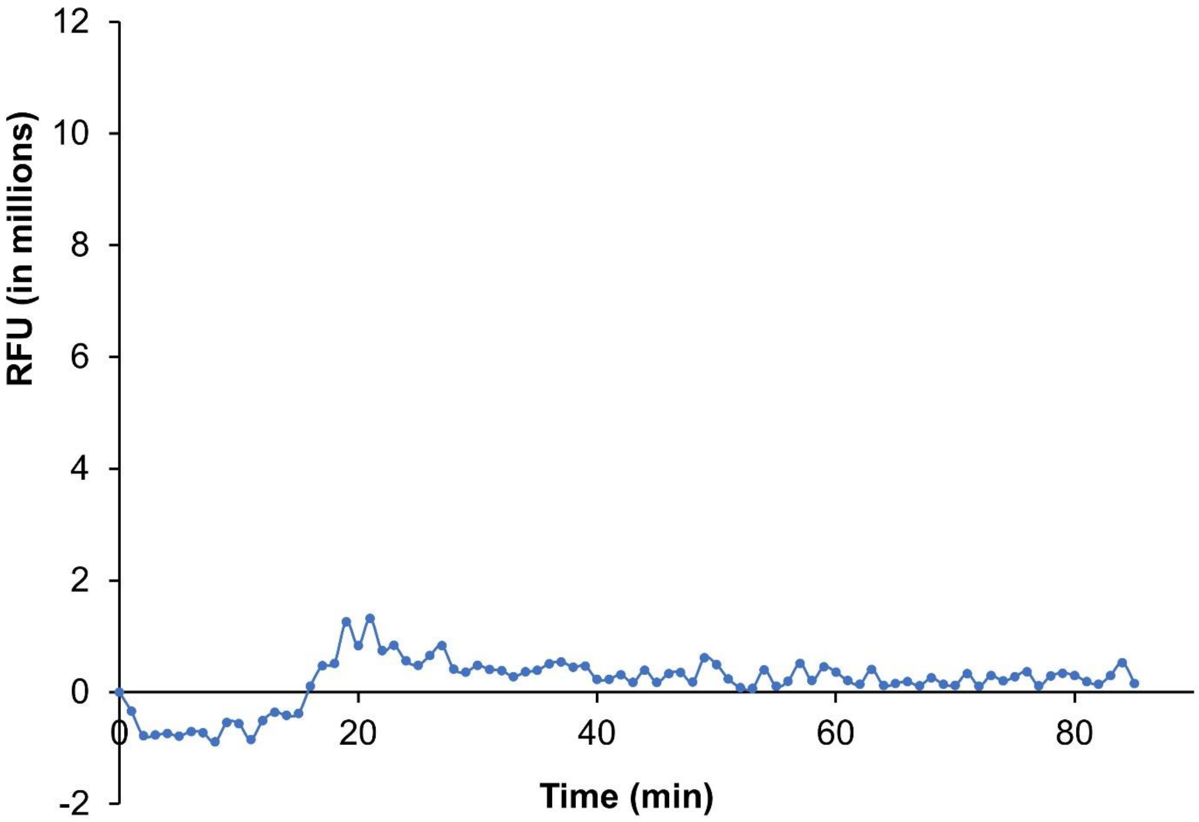

Figure S8 **CP-N does not bear any cGAMP production activity as shown by the fluorescence-based assay.** RFU: relative fluorescence unit.
