# Supplementary material for: The Dengue virus protease NS2B3 cleaves cyclic GMP-AMP synthase to suppress cGAS activation
Source: J Biol Chem. 2023 Feb 7;299(3):102986. doi: 10.1016/j.jbc.2023.102986 (PMC10011430; doi:10.1016/j.jbc.2023.102986)
Supplement: Supporting Tables S2–S6 [file mmc3.docx]

| ***K*_d_ (nM)** | FL | CP-C | CP-N |
| --- | --- | --- | --- |
| Measurement 1 | 71 ± 5 | 238 ± 23 | 188 ± 37 |
| Measurement 2 | 72.5 ± 5 | 172 ± 10 | 151 ± 32 |

**Table S2** Summary of DNA binding affinities between ISD45 and FL cGAS, CP-C, or CP-N fragments as measured by fluorescence polarization (FP). Measurement 1 data is plotted in Figure 3B.

| ***K*m (μM)** | FL | CP-C |
| --- | --- | --- |
| Measurement 1 | 274.7 ± 39.3 | 224.5 ± 66.7 |
| Measurement 2 | 282.6 ± 15.9 | 225.8 ± 69.2 |
| Measurement 3 | 242.4 ± 83.1 | 235.1 ± 65.6 |

**Table S3** Summary of Km values of FL cGAS and CP-C. Measurement 1 data are plotted in Figure S7.

| **Protein Batches** | **Fluorescence Assays** | **IC50 (μM)** |
| --- | --- | --- |
| Prep 1 | Experiment 1 | 19.6 ± 4 |
|  | Experiment 2 | 25.7 ± 2.5 |
| Prep 2 | Experiment 3 | 20.4 ± 3.5 |
| Prep 3 | Experiment 4 | 4.7 ± 0.8 |
|  | Experiment 5 | 13.9 ± 4 |

**Table S4** Summary of IC50 values of CP-N inhibition of FL cGAS activity. Three independent batches of proteins were used; and one or two fluorescence-based assays were carried out with each batch. Experiment 5 data are plotted in Figure 4A and 4C.

| **Protein Batches** | **Fluorescence Assays** | **IC50 (μM)** |
| --- | --- | --- |
| Prep 1 | Experiment 1 | 21.7 ± 1.7 |
|  | Experiment 2 | 32.3 ± 3.2 |
| Prep 2 | Experiment 3 | 36.5 ± 9 |

**Table S5** Summary of IC50 values of CP-N inhibition of CP-C activity. Two independent batches of proteins were used; and one or two fluorescence-based assays were carried out with each batch. Experiment 2 data are plotted in Figure 4B and 4D.

|  | **Protein Batches** | ***K*_d_ (μM)** |
| --- | --- | --- |
| FL cGAS | Prep 1 | 21.6 ± 2.9 |
|  | Prep 2 | 27.1 ± 17.8 |
| CP-C | Prep 1 | 39.2 ± 2.4 |
|  | Prep 2 | 42.0 ± 4.2 |

**Table S6** Summary of binding affinities between FL cGAS or CP-C and NS2B3^S135A^ as assessed by the gel shift assay in Figure 5. Two independently prepared proteins were used for the gel shift assay.
